# Incidence and Health Care Burden of Uterine Fibroids Among Female Service Members in the Active Component of the U.S. Armed Forces, 2011–2022

**Published:** 2024-02-20

**Authors:** Chiping Nieh, Sithembile L. Mabila

**Affiliations:** 1 Epidemiology and Analysis Branch, Armed Forces Health Surveillance Division, Defense Health Agency

## Abstract

Uterine fibroids are the most common benign tumors of the uterus among women of reproductive age, disproportionally affecting non-Hispanic Black women compared to other races and ethnicities. This report is an update of a 2011 *MSMR* report that examined uterine fibroids among female active component service members in the U.S. Armed Forces from 2001 to 2010. Incident uterine fibroids were identified for this report from inpatient and outpatient medical encounter data from 2011 to 2022. Health care burden was estimated utilizing uterine fibroid-related inpatient and outpatient diagnostic and procedure codes. Crude incidence rates and incidence rate ratios were calculated to compare rate differences between subpopulations. A total of 16,046 new uterine fibroid cases were identified, with an incidence rate of 63.5 cases per 10,000 person-years (95% confidence interval: 62.5-64.5). The highest incidence rates were observed among service women 40 years and older, non-Hispanic Black women, and those who served in the Army. Health care burden analysis showed that, even with increases in medical encounters and individuals affected, the numbers of hospital bed days declined over time. The decline in uterine fibroid-related hospital bed days could be attributed to early diagnoses and minimally-invasive treatments. Continued promotion of uterine fibroid awareness can potentially help further reduce uterine fibroidrelated impacts on military readiness.

**What are the new findings?:**

Uterine fibroid-related medical encounters and individuals affected increased over time from 2011 to 2022, but the number of hospital bed days decreased from 699 days in 2011 to 625 days in 2022. This decrease in bed days could be attributed to the early detection of uterine fibroid cases and increased accessibility of non- or less invasive treatments.

**What is the impact on readiness and force health protection?:**

With the growth of medical encounters and individuals affected over time, the decline in uterine fibroid-related hospital bed days shows that early diagnoses and minimally-invasive treatments can effectively reduce uterine fibroid-related health care burdens and minimize impacts on military readiness.

## 1 BACKGROUND

Uterine fibroids are the most common benign tumors of the uterus among women of reproductive age, disproportionally affecting Black women compared to other races and ethnicities.^1,2^ A systematic review by Guiliani et al. found high prevalence of uterine fibroids detected by ultrasound among U.S. Black and White women, 80% and 70% respectively, by the age of 50.^2^ Guiliani et al. reported that Black women were more likely to have uterine fibroids at younger ages, or larger fibroid sizes, compared to their counterparts. In a more recent study, Huang et al. found that, besides Black women, Asian-Chinese women were also disproportionately affected by uterine fibroids.^3^

In addition to race and ethnicity, other risk factors of uterine fibroids reported in the literature include older age, early menarche, obesity, stress, hypertension, nulliparity, late menopause, and family history.^2,4,5,6^ Many studies have focused on the disproportionally high incidence of uterine fibroids among Black women and identified potential risk factors including lack of fruit, vegetable or fiber intake, vitamin D deficiency, obesity, life stressors, in addition to limited access to care.^7,8,9^

Approximately 30% of women with uterine fibroids experience symptoms such as profound bleeding, anemia, pelvic pressure or pain, in addition to others.^2^ Many different treatments are available for symptomatic uterine fibroids.^10^ Recommended treatment for each patient is determined based upon the number, size, and location of tumors, as well as the patient’s desire for maintaining fertility.^11^ Recently, with growing interest among women for non-surgical treatments, more minimally-invasive methods, including uterine artery embolization, high intensity focused ultrasound, microwave ablation, and radiofrequency ablation, have become available.^12^

Due to the prevalence of uterine fibroids and advanced treatments associated with it, annual direct and indirect costs in the U.S. are estimated to be as high as $34.4 billion.^2^ In addition to health care costs, symptomatic uterine fibroids can also cause loss of work days or work productivity.^13^

Regardless of the high prevalence and potential impacts of uterine fibroids, women have continued to delay seeking medical attention due to lack of knowledge or awareness.^14^ During the past 10 years, researchers began examining any associations between hair products used by Black women and their risk of developing uterine fibroids or uterine cancer,^15,16^ which helped raise uterine fibroid awareness. Marsh et al.^11^ found, however, that only half of women experiencing fibroid-related symptoms without clinical diagnosis had heard of uterine fibroids. Marsh et al. also found that lack of awareness was more prevalent among low-income women or racial minorities. Since many uterine fibroids are discovered by chance, through routine pelvic exams,^4^ continuing to raise awareness is important, for encouraging women who are experiencing symptoms to consult health care providers.

This report is an update of a 2011 *MSMR* report,^17^ published as a Surveillance Snapshot, that provided numbers, rates, and demographic characteristics of uterine fibroids as well as uterine fibroid-related health care among female service members in the active component of the U.S. Armed Forces. The 2011 report identified higher rates of uterine fibroids among non-Hispanic Black women among all age groups.^17^ The 2011 report also showed a declining trend of hysterectomy treatments among patients.

This report aims to confirm whether similar trends to those discerned in 2011 persisted from 2011 to 2022 among female service members. This report also assesses possible procedural delays due to the coronavirus disease (COVID-19) pandemic related to new gynecological ultrasound guidance published by the International Society of Ultrasound in Obstetrics and Gynecology,^18^ following COVID-19 guidelines for non-emergent surgical procedures developed by the American College of Surgeons.^19^ During the COVID-19 pandemic, health care providers were more likely to offer fibroid patients less invasive procedures that could be performed in an outpatient setting or required no more than 1 overnight hospital stay.^20^

## 2 METHODS

The study population of this report included all female service members serving in the active component of any branch of the U.S. Armed Forces during the surveillance period, from January 1, 2011 through December 31, 2022. Data were queried from the Defense Medical Surveillance System (DMSS). Uterine fibroid cases were determined based on outpatient and inpatient administrative health records from both direct and purchased care.

**Case definition**

International Classification of Diseases, 9th/10th revision (ICD-9/ICD-10) diagnostic codes were used to define uterine fibroid cases (**Table 1**). Female service members with either 1 inpatient or outpatient medical encounter with a uterine fibroid case-defining code in the primary diagnostic position, or a case-defining code in the secondary diagnostic position paired with an associated symptom code in the primary diagnostic position, were considered as uterine fibroid cases. The date of the first case-defining inpatient or outpatient medical encounter was used as the incident date, and service members were counted once per lifetime. Service members with a case-defining encounter prior to the surveillance period were deemed ineligible and excluded from this report.

**Health care burden**

To precisely measure health care burdens associated with uterine fibroids, only inpatient or outpatient medical encounters with a case-defining code in the primary diagnostic position were included. Uterine fibroid-related procedures were identified using ICD-9/ICD-10 procedural codes as well as the Current Procedural Terminology (CPT) codes (**Table 1**). Procedures were then categorized into 3 groups: Hysterectomy, Myomectomy, and Other. Total numbers of medical encounters, individuals affected, hospital bed days, as well as numbers and percentages of uterine fibroid-related treatment procedures were utilized to quantify the health care burdens of uterine fibroids in the U.S. Armed Forces.

**Time-censoring calculations**

For each eligible service member’s person-time calculations, the most recent of 2 dates—either military enrollment or the beginning of the surveillance period, January 1, 2011—was used as the starting time. Time was then censored at the earliest of 3 dates: date of uterine fibroid diagnosis, date of separation from active component or military service, or end of the surveillance period, December 31, 2022.

**Statistical analysis**

Incident cases of uterine fibroids by key demographic variables were determined. Crude incidence rates (IRs) were calculated as incident uterine fibroid diagnoses per 10,000 person-time (p-years) with 95% confidence intervals (CIs). Based on the IRs, the least at-risk subgroup of each demographic variable was selected as the reference group. Incidence rate ratios (IRRs) were then calculated. All analyses were conducted using SAS Enterprise Guide (version 8.3).

## 3 RESULTS

The study population included female service members who served in the active component, from January 1, 2011 through December 31, 2022. Among 586,252 eligible female service members, a total of 16,046 new onset uterine fibroid cases were identified, for an IR of 63.5 per 10,000 p-years (95% CI: 62.5-64.5). Among all new onset cases, 97% (n=15,578) were outpatient cases.

As shown in **Table 2**, highest incidence rates of uterine fibroids were observed among service women aged 40 years and older (IR: 276.6 per 10,000 p-yrs, 95% CI: 268.9-284.2), non-Hispanic Black women (IR: 150.5 per 10,000 p-yrs, 95% CI: 147.3-153.6), and those serving in the Army (IR: 85 per 10,000 p-yrs, 95% CI: 83-87). Incidence rate ratio analysis revealed that non-Hispanic Black women were almost 5 times more likely to develop uterine fibroids than their non-Hispanic White counterparts. Service women aged 40 years and older were 29 times more likely to be diagnosed with uterine fibroids than those under 25 years of age (**Table 2**). **Table 2**) also demonstrates a trend of increasing uterine fibroids with increased ages of service women.

**Figure 1:**
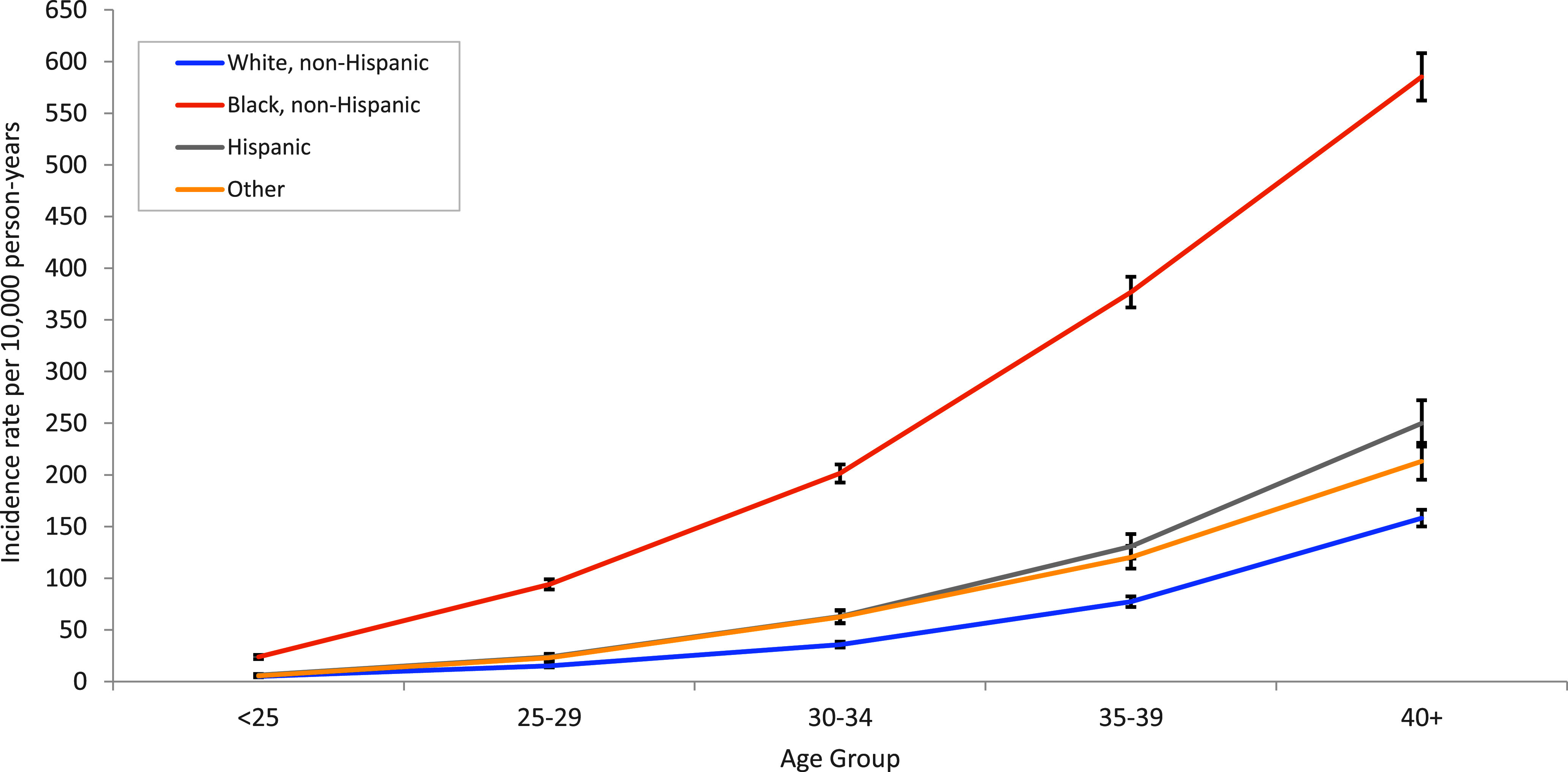
Incidence Rates of Uterine Fibroids by Age and Race or Ethnicity Among Active Component Female Service Members, 2011–2022

When examining uterine fibroid cases by both age and race and ethnicity, incidence rates of uterine fibroids among non-Hispanic Black service women were consistently higher than among other races and ethnicities, for all age groups (**Figure 1**). The incidence rate differences between non-Hispanic Black women and other races and ethnicities augmented with increasing age.

**Figure 2:**
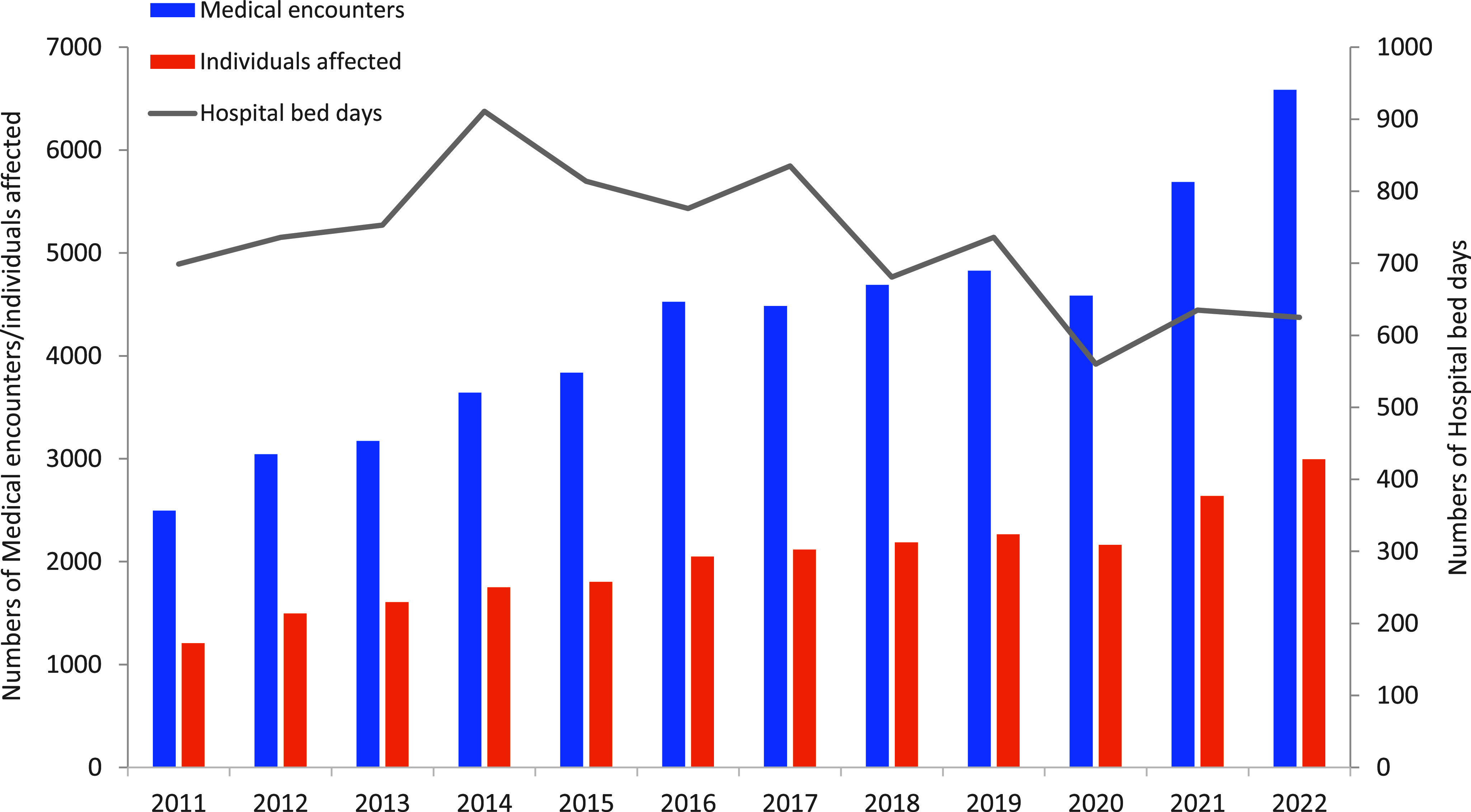
Total Numbers of Uterine Fibroid-related Medical Encounters, Individuals Affected, and Hospital Bed Days Among Active Component Female Service Members, 2011–2022

As shown in **Figure 2**, health care burden analysis revealed that both numbers of medical encounters and individuals affected increased over time, with medical encounters showing a steeper upward slope. From 2011 to 2022, annual numbers of medical encounters increased from 2,496 to 6,585, while numbers of individuals affected rose from 1,208 to 2,994, with a slight decrease of both medical encounters and individuals affected in 2020, followed by elevations in 2021 and 2022. During the surveillance period, the total number of female service members also increased substantially, from 193,211 in 2011 to 228,145 in 2022. Regardless of the increase of both medical encounters and individuals affected, the total number of bed days declined gradually after peaking at 911 days in 2014, from 699 days in 2011 to 625 days in 2022.

**Figure 3:**
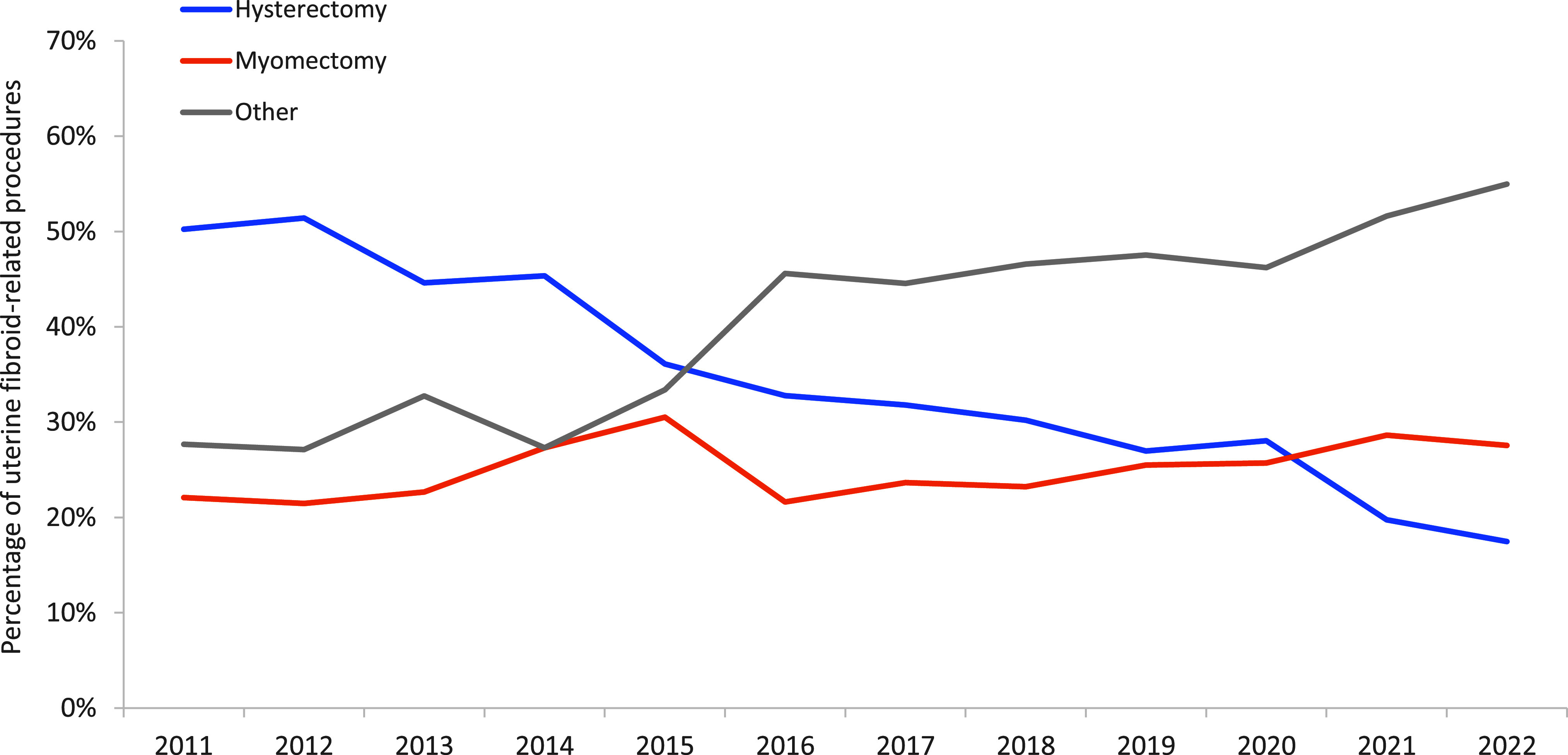
Percentages of Selected Medical or Surgical Procedures Among Uterine Fibroid Cases with First Listed Code as a Case-defining Code, 2011–2022

The percentage of hysterectomies declined consistently, from 50% in 2011 to 17% in 2022 (**Figure 3**). While fibroid-related myomectomy treatments trended upward over the surveillance period, from 22% to 28%, the speed of the increase was relatively low. A higher speed of increase was observed (from 28% to 55%) among other fibroid-related treatments including uterine artery embolization (UAE) and hysteroscopy dilation and curettage (D&C, hysteroscopy) (data not shown).

## 4 DISCUSSION

This report summarizes counts, incidence rates, and treatment trends of uterine fibroids among female service members in the active component of U.S. Armed Forces from 2011 to 2022. The same pattern of highest incidents among older non-Hispanic Black female service members reported in the 2011 report^17^ was observed in this analysis. This report also found non-Hispanic Black women more likely to develop uterine fibroids at a younger age, confirming existing literature.^2,4,17^

Potential associations between uterine fibroids and Black women include use of certain hair products, insufficient nutrition, vitamin D deficiency, obesity, life stressors, in addition to limited access to care.^7,8,9,15^ While the chance of obesity in the military is low, and all service members have access to health care, individual lifestyle factors such as hair product use and fruit, vegetable and vitamin consumption among Black female service members is likely comparable to their civilian counterparts. Additionally, the military is regarded as a stressful work environment. These associated factors may contribute to similar patterns in the military population.

Among all incident cases identified from 2011 to 2022, 97% were outpatient cases, which was elevated from the 92% outpatient cases in the 2011 *MSMR* report.^17^ This result could be attributed to increased public awareness of uterine fibroids^14,15,16^ that resulted in more women with mild symptoms seeking medical diagnoses during outpatient visits.

During the surveillance period, both medical encounters and numbers of individuals affected increased gradually, following similar trends observed in the 2011 report.^17^ The current report did observe a slight decrease, however, of medical encounters and individuals affected in 2020, followed by elevations in 2021 and 2022. That slight decrease could be attributed to the overall decline in primary care due to overwhelmed and strained health care systems during the COVID-19 pandemic.

Despite the increase of medical encounters and individuals affected over time, in this report hospital bed days showed a consistent downward trend, differing from the 2011 report.^17^ This report also identified a significant decline in invasive procedures, and an increase in other procedures, which explain the reduction in hospital bed days. It is possible that early detection of uterine fibroid cases combined with access to non- or less invasive treatments such as UAE and D&C, hysteroscopy have reduced uterine fibroid-related hospital bed days over time. A more rapid decrease of hospital bed days was also observed in 2019-2020, which could be attributed to procedural delays or the preference of less invasive procedures during the COVID-19 pandemic.^18,20^

This report has some limitations. The severity of uterine fibroid cases could not be categorized due to lack of specific medical information such as tumor size, location, and numbers. Additionally, DOD Instruction 6130.0321 defines history of chronic pelvic pain and abnormal uterine bleeding as accession-limiting. As a result, the data for those under 25 years old could be skewed. Lastly, the case definition did not include individuals with a diagnosis of abnormal uterine bleeding if there was no diagnosis of uterine fibroids in the primary or secondary diagnostic position. Consequently, use of this more specific case definition may have underestimated incidence of uterine fibroids within this population.

This report reassessed trends last reported in 2011, to provide an updated general assessment of the impact of uterine fibroids among female service members in the active component of the U.S. Armed Forces. Additionally, this report examined potential COVID-19-associated procedural delays in treatment. While the total number of cases during the surveillance period increased over time, a concurrent reduction of hospital bed days demonstrates a better approach for disease management, contributing to reduced costs of uterine fibroid-related medical care and lost work days.

Even with universal health care access, non-Hispanic Black service women continue to be disproportionally affected by uterine fibroids. This finding indicates that the higher incidence of uterine fibroids among non-Hispanic Black women are not primarily caused by health care accessibility. Additional, well-designed research is needed to further examine any genetic, behavioral, or environmental risk factors for uterine fibroids among this subpopulation.

## Figures and Tables

**Table 1.** Uterine Fibroids Case-defining Codes, Associated Symptom Codes, and Uterine Fibroid-related Inpatient and Outpatient Procedure Codes

	**ICD-10**	**ICD-9**	**CPT**
**Case-defining codes**			
Uterine leiomyomas (fibroids)	D25.-	218.xx	
			
**Associated symptom codes**			
Anemia due to blood loss	D50.0, D62	280.0, 285.1	
Vaginal bleeding, menstrual bleeding disorders	N89.8, N92.0, N92.1, N92.3, N92.4, N92.5, N92.6, N93.8, N93.9	623.8, 626.2, 626.6, 626.5, 627.0, 626.8, 626.9	
			
**Inpatient procedure codes**			
Hysterectomy	0UT97ZL, 0UT97ZZ, 0UT98ZL, 0UT98ZZ, 0UT90ZL, 0UT90ZZ, 0UT94ZL, 0UT94ZZ, 0UT9FZL, 0UT9FZZ	683, 6831, 6839, 684, 6841, 6849, 6851, 6859, 686, 6879, 689	
Myomectomy	0UB90ZZ	6829	
			
**Outpatient procedure codes**			
Hysterectomy			58150, 58152, 58180, 58200, 58210, 58951, 58953, 58954, 59525, 56308, 58550, 58552, 58553, 58554,58260, 58261, 58262, 58263, 58267, 58270, 58275, 58280, 58285, 58290, 58291, 58292, 58293, 58294
Myomectomy			58140, 58145, 58146, 58545, 58551, 58546
D&C, hysteroscopy			58120, 58555, 58558, 58561, 58563
Uterine artery embolization			37204
Other (endometrial ablation)			58353
Magnetic resonance-guided focused ultrasound surgery			0398T, 0071T, 0072T

Abbreviation: D&C, Dilation and curettage

**Table 2.** Numbers and Rates of Uterine Fibroids by Demographics and Miltiary Characteristics Among Active Component Female Service Members, 2011–2022

**Characteristic**	**No. of Cases**	**IR^a^ (95% CI)**	**IRR (95% CI)**
Total	16,046	63.5 (62.5, 64.5)	---
**Age group**			
<25	1,000	9.6 (9.0, 10.1)	Ref
25-29	2,329	36.2 (34.7, 37.7)	3.8 (3.5, 4.1)
30-34	3,429	85.2 (82.3, 88.0)	8.9 (8.3, 9.6)
35-39	4,301	169.3 (164.3, 174.4)	17.7 (16.5, 19.0)
40+	4,987	276.6 (268.9, 284.2)	29.0 (27.1, 31.0)
			
**Inpatient procedure codes**			
**Race and ethnicity**			
White, non-Hispanic	3,648	32.3 (31.3, 33.4)	Ref
Black, non-Hispanic	8,974	150.5 (147.3, 153.6)	4.7 (4.5, 4.8)
Hispanic	1,727	38.9 (37.1, 40.7)	1.2 (1.1, 1.3)
Other	1,697	47.4 (45.1, 49.6)	1.5 (1.4, 1.6)
			
**Service**			
Army	6,986	85.0 (83.0, 87.0)	4.1 (3.7, 4.5)
Navy	3,471	48.4 (46.8, 50.1)	2.3 (2.1, 2.6)
Air Force	4,785	64.3 (62.5, 66.2)	3.1 (2.8, 3.4)
Marine Corps	373	20.8 (18.7, 22.9)	Ref
Coast Guard	431	65.4 (59.3, 71.6)	3.1 (2.7, 3.6)

Abbreviations: No., number; IR, incidence rate; CI, confidence interval; IRR, incidence rate ratio.

^a^per 10,000 person-years

## AUTHOR AFFILIATIONS

Epidemiology and Analysis Section, Armed Forces Health Surveillance Division, Defense Health Agency, Silver Spring, MD: Dr. Nieh, Dr. Mabila
